# Reciprocal Effects between Sleep Quality and Life Satisfaction in Older Adults: The Mediating Role of Health Status

**DOI:** 10.3390/healthcare11131912

**Published:** 2023-07-01

**Authors:** Change Zhu, Lulin Zhou, Xinjie Zhang, Christine A. Walsh

**Affiliations:** 1 Department of Management, Jiangsu University, 301 Xuefu Road, Jingkou District, Zhenjiang 212001, China; 2 Faculty of Social Work, University of Calgary, 2500 University Drive NW Calgary, Calgary, AB T2N 1N4, Canada

**Keywords:** reciprocal effects, sleep quality, life satisfaction, health status, cross-lagged panel analysis

## Abstract

Objectives: to examine the causal relationship between sleep quality and life satisfaction and explore the mediating role of health status on the relationship between sleep quality and life satisfaction. Methods: A total of 1856 older Chinese people participating in 2011, 2014, and 2018 waves of the Chinese Longitudinal Healthy Longevity Survey (CLHLS) were included. A cross-lagged panel analysis (CLPA) combined with mediator analysis was utilized. Results: The average sleep quality levels for the years 2011, 2014, and 2018 were 3.70, 3.63, and 3.47 out of 5, respectively. The corresponding average levels of health status were 3.47, 3.44, and 3.39 out of 5, and the average levels of life satisfaction were 3.75, 3.86, and 3.87 out of 5, respectively. In addition, sleep quality at prior assessment points was significantly associated with life quality at subsequent assessments, and vice versa. Also, health status partially mediated this prospective reciprocal relationship. Conclusions: There is a nonlinear decreased trend in sleep quality and health status, while there exists a nonlinear increased trend in life satisfaction for older adults from 2011 to 2018. Reciprocal positive effects between sleep quality and life satisfaction in older adults exist and are mediated by health status.

## 1. Background

The aging population in China is rapidly increasing. According to data released by the National Bureau of Statistics of China in 2021, the older population aged 60 and above reached over 267 million people (data source: http://www.stats.gov.cn/sj/ndsj/2022/indexch.htm, accessed on 7 June 2023). With advancements in medical care, nutrition, and living standards, the average life expectancy of the elderly is increasing, further deepening the aging trend. According to projections, the population of individuals aged 65 years or older is expected to more than double by the year 2050, reaching an estimated 366 million, which would represent 26.1% of the total population (data source: UN. World population prospects 2019. https://population.un.org/wpp, accessed on 7 June 2023). This trend poses challenges and opportunities for addressing the impact of aging on China’s economy and society. Due to the increasing population in this age group, the quality of life of these individuals has become a matter of concern. Life satisfaction is commonly considered to be an important predictor of quality of life [1] and successful aging [2,3]. Life satisfaction refers to a broad and profound sense of happiness that arises from one’s experiences in the external world. It represents an individual’s positive outlook on life and reflects their emotions related to their past, present, and future [4].

Life satisfaction is generally correlated with various demographic variables, for instance, gender [5], educational attainment [6], marital status [7], and living arrangements [8,9]. Furthermore, life satisfaction was suggested to be linked with financial strain [10], depressive symptoms [5], filial piety [11], family support [12], and accessibility of health services [13,14] for older adults. Recent evidence indicates that sleep quality is a predictor of life satisfaction among older adults, such that the better the sleep quality, the higher the life satisfaction [15,16].

Sleep quality is a crucial aspect of daily life for older adults. For example, it was reported that nearly 50% of older adults were experiencing at least one chronic sleep-related issue, with the most prevalent being difficulty in maintaining sleep during the night [17]. A study on older residents of urban China showed that the prevalence of poor sleep quality was 41.5%, with a higher incidence among women than men. Risk factors for poor sleep quality were identified as being age, low educational attainment, living alone, anxiety, multiple chronic diseases, and arthritis [18]. There is substantial evidence from prior research indicating that inadequate sleep, as identified via self-reported sleep quality, limited duration of sleep, and presence of a sleep disorder are linked with a broad range of health risks, such as elevated body weight, cardiovascular illness, and overall mortality [19,20,21,22].Therefore, more attention should be paid to the sleep quality of older adults and examining the change trajectory in sleep quality.

Additionally, sleep is an integral component of a healthy lifestyle and plays a vital role in maintaining overall health status [16]. As mentioned above, poor sleep quality is correlated with health risks, indicating that poor sleep quality will result in a decreased health status. Also, health status is demonstrated to be a predictor of life satisfaction [7]. To put it differently, older individuals who maintain good health also tend to have higher levels of life satisfaction [23]. Therefore, the aforementioned argument suggests that the relationship between sleep and life satisfaction is mediated by one’s health status.

However, studies have also demonstrated that life satisfaction has an impact on sleep quality [24,25,26]. Therefore, it is necessary to analyze whether there exists a bidirectional relationship between sleep quality and life satisfaction. Moreover, life satisfaction can also affect perceived health status [27,28], which was suggested to be linked with sleep quality [29]. Therefore, health status is assumed to mediate the relationship between life satisfaction and sleep quality.

To sum up, the association between sleep quality and life satisfaction has garnered considerable attention. As previously noted, there is a positive correlation between sleep quality and life satisfaction, and life satisfaction, in turn, has been linked to sleep quality. This suggests a bidirectional relationship between the two. Nonetheless, there is a paucity of research that specifically examines the causal relationship between sleep quality and life satisfaction. Moreover, extant studies about the relationship between sleep quality and life satisfaction are based on cross-sectional studies, which cannot accurately infer causal relationships between variables.

Therefore, utilizing longitudinal national representative data, the article aims (1) to examine the change trajectory in the sleep quality, life satisfaction, and health status of older adults from 2011 to 2018; (2) to explore the bidirectional relationship between sleep quality and life satisfaction; and (3) to analyze the mediating effect of health status in the bidirectional relationship between sleep quality and life satisfaction.

## 2. Methods

### 2.1. Data Sources

**Figure 1:**
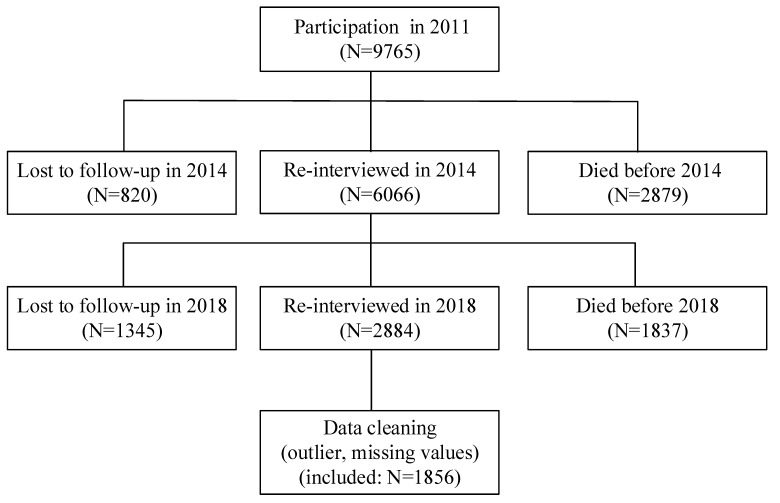
Flowchart of data source.

The data used in this study were obtained from the Chinese Longitudinal Health Longevity Study (CLHLS), which is a dynamic cohort study [30]. The initial data were collected in 1998, and subsequently, seven follow-up surveys were conducted in 2000, 2002, 2005, 2008, 2011, 2014, and 2018, respectively. This survey is a comprehensive longitudinal study conducted across 23 provinces/autonomous regions in China. The regions were selected randomly for in-person household interviews. The survey encompassed a wide range of basic information regarding the older population’s dietary habits, behavior and lifestyle, and disease status. The main objective of this article was to investigate the reciprocal relationship between sleep quality and life satisfaction among older adults. Additionally, this study also explores the mediating effect of health status between sleep quality and life satisfaction. The data utilized in this article were sourced from the latest three waves of the CLHLS conducted in 2011, 2014, and 2018. To ensure the comprehensive tracking of changes in the quality of sleep and life satisfaction among the older adults, this research included individuals who participated in the 2011, 2014, and 2018 surveys. Ultimately, a total of 1856 valid older individuals were identified after data cleaning. The details of the data source are shown in Figure 1.

### 2.2. Variable Definitions

Sleep quality. Sleep quality was measured by asking the question “how about the quality of your sleep?”, with a score of 1 indicating very poor and 5 indicating very good. The higher the score, the better the quality of sleep. A single-item self-report measure of sleep quality has been widely used in previous studies [31,32].

Health status. Health status was measured by asking the question “how do you rate your health at present?”. On a scale of 1 to 5, with 1 indicating very poor and 5 indicating very good, the higher the score, the higher the health status. A single-item self-report health status has been commonly used in previous studies and is suggested to be a good indicator of older adults’ real health status [32].

Life satisfaction. Life satisfaction was measured by asking the question “how do you rate your life at present?”, with a score of 1 representing very bad and 5 representing very good. The higher the score, the higher the level of life satisfaction. The question is considered to be a general indicator of assessing life satisfaction [33] and is reported to have good reliability and validity [34].

The study included a range of demographic variables such as age, gender, region, education, marital status, and living arrangement. In addition, several covariates were included, such as social security (retirement pension, public free medical services, collective medical insurance for urban residents, etc.), smoking and alcohol drinking habits, physical exercise, and cognitive ability. Cognitive ability was assessed using a comprehensive set of 24 items, covering general ability (3 items), responsiveness (3 items), attention and calculation ability (6 items), recall (3 items), and language comprehension and self-coordination (6 items). The responses for each item were recorded on a dichotomous scale, where 1 indicates a correct response and 0 indicates a wrong response. However, for the item “number of kinds of food named in 1 min” the maximum score was 7. Therefore, the overall range for cognitive ability was 0-30. More detailed information regarding these variables is available in Table 1.

### 2.3. Data Analysis

In this study, descriptive statistical analysis was used to analyze the sample information of older people. In addition, the advantages of CLPA lie in its ability to establish temporal precedence, assess causal relationships, control for stability, and mitigate methodological biases [35]. Thus, using MPLUS 8.3 software (Linda Muthén & Bengt Muthén, Los Angeles, CA, USA), a cross-lagged panel analysis (CLPA) was employed to investigate the reciprocal association between sleep quality and life satisfaction among older adults in 2011, 2014, and 2018, with covariates controlled. Furthermore, the study explores the mediating effect of the health status of the older population on the reciprocal relationship between sleep quality and life satisfaction.

## 3. Results

### 3.1. Descriptive Analysis of the Sample

According to Table 1, it can be observed that the average age of older adults is 80.52, with males accounting for 52% and 92% of the older population coming from rural areas. Sixty-two percent of the older people are married, and overall, the educational level of older adults is low, with an average of 3.11 years of education. In addition, the percentage of older adults who smoke and drink is 35% and 30%, respectively. Thirty-five percent of older adults exercise regularly. Regarding social security, older adults have an average of 1.37 social security items. Finally, the average level of cognitive ability of older adults is 26.38. The mean levels of sleep quality, health status, and life satisfaction are 3.60, 3.43, and 3.83, respectively. The details for each wave are shown in Table 2.

### 3.2. Trajectory of Sleep Quality, Life Satisfaction, and Health Status of Older Adults

**Figure 2:**
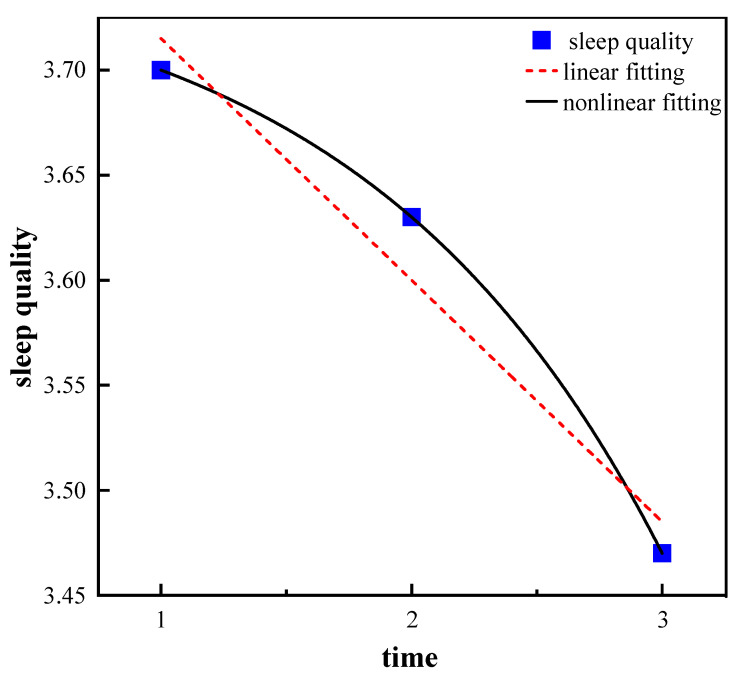
Trajectory of sleep quality.

**Figure 3:**
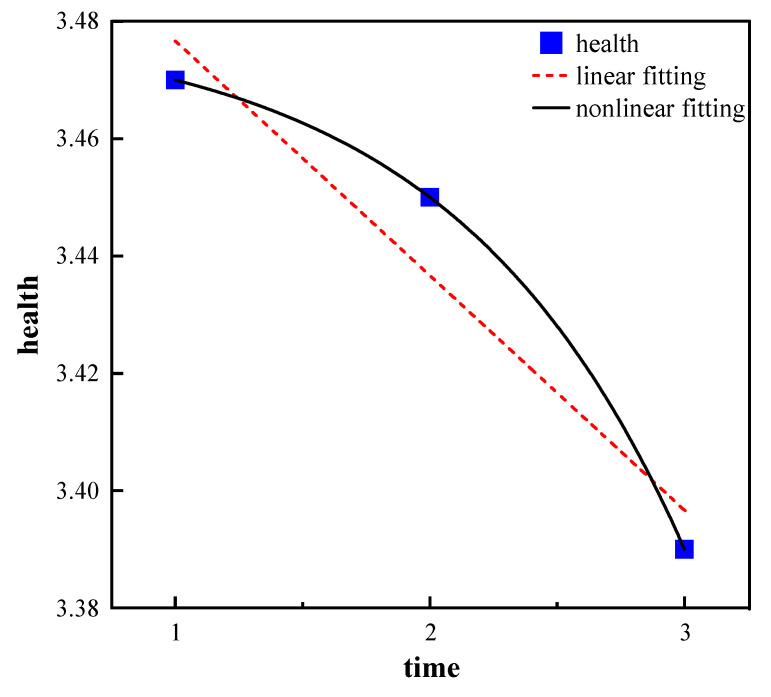
Trajectory of health status.

**Figure 4:**
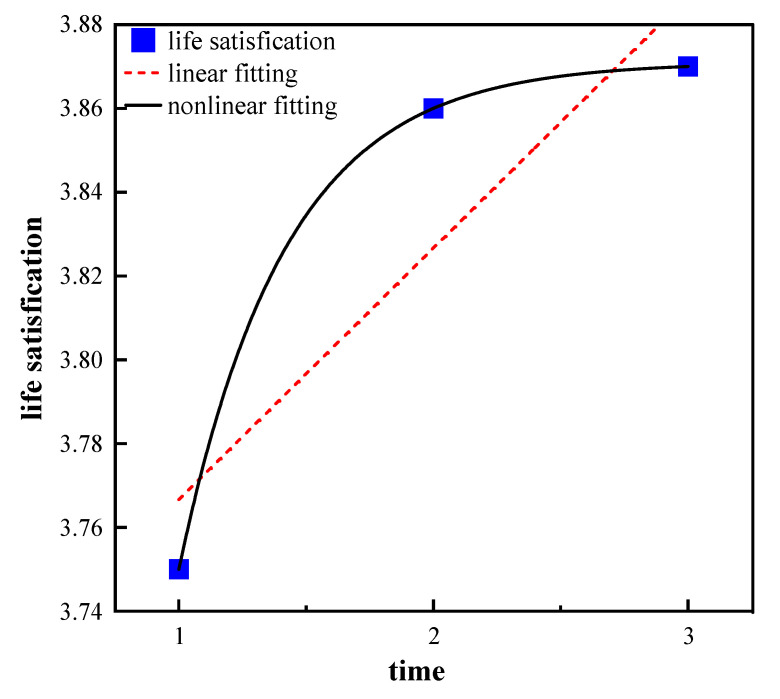
Trajectory of life satisfaction.

According to Table 2, the sleep quality scores were 3.70, 3.63, and 3.47 in 2011, 2014, and 2018, respectively. The life satisfaction scores were 3.75, 3.86, and 3.87, while the health status scores were 3.47, 3.44, and 3.39 for each of the CLHLS waves. Linear fitting and nonlinear fitting were both employed to analyze the change trajectories in sleep quality, health status, and life satisfaction, and the results showed that the nonlinear fitting performed better than the linear fitting for these variables. The above results suggest that older adults experienced a quadratic growth trend in life satisfaction, and a decreasing trend in sleep quality and health status from 2011 to 2018, as indicated in Figure 2, Figure 3 and Figure 4.

### 3.3. Reciprocal Relationship between Sleep Quality and Life Satisfaction

As depicted in the background, there may a reciprocal relationship between sleep quality and life satisfaction. To further examine the real relationship between them, a cross-lagged panel model was constructed. Over time, changes in individuals can stem from both within-individual differences and between-individual differences. Thus, it is essential to account for the influence of age or time in longitudinal studies in order to control for these factors [36].

**Figure 5:**
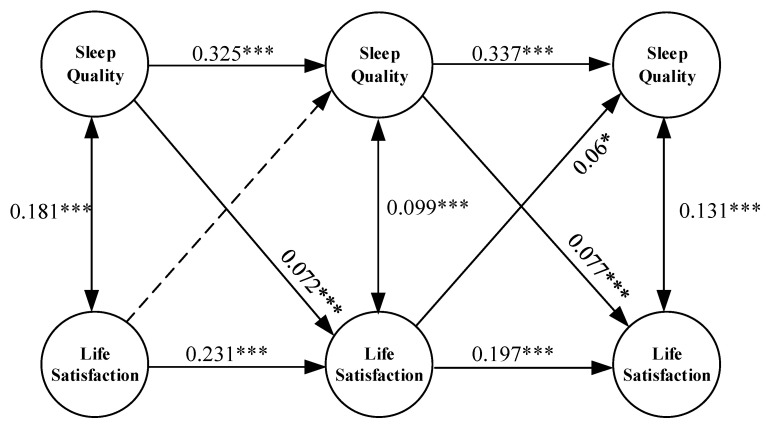
Cross-lagged panel model of reciprocal relationship between sleep quality and life satisfaction. Footnote: *** denotes *p* < 0.001

As shown in Figure 5, the model fits well after the introduction of control variables (age, gender, and marriage) and covariates (physical exercise, social security, and cognitive ability), with fitting indicators of RMSEA = 0.048, CFI = 0.848, SRMR = 0.043, and *p* = 0.000. To be specific, the sleep quality of older adults in 2011 had a significant positive impact on both their life satisfaction and sleep quality in 2014, as indicated by the values of B = 0.072 and *p* = 0.000 for life satisfaction, and B = 0.325 and *p* = 0.000 for sleep quality. However, life satisfaction in 2011 did not significantly affect sleep quality in 2014. On the other hand, life satisfaction in 2011 had a significant positive effect on life satisfaction in 2014, with B = 0.231 and *p* = 0.000. Additionally, sleep quality in 2014 had a significant correlation with both life satisfaction and sleep quality in 2018, with B = 0.077 and *p* = 0.000 for life satisfaction, and B = 0.337 and *p* = 0.000 for sleep quality. Furthermore, life satisfaction in 2014 had a significant positive effect on both sleep quality and life satisfaction in 2018, as indicated by B = 0.06 and *p* = 0.034 for sleep quality, and B = 0.197 and *p* = 0.000 for life satisfaction. Finally, it is worth noting that sleep quality and life satisfaction were significantly positively correlated in 2011, 2014, and 2018, with r = 0.181 ***, r = 0.099 ***, and r = 0.131 ***, respectively.

### 3.4. The Mediating Effect of Health Status in the Bidirectional Relationship between Sleep Quality and Life Satisfaction

**Figure 6:**
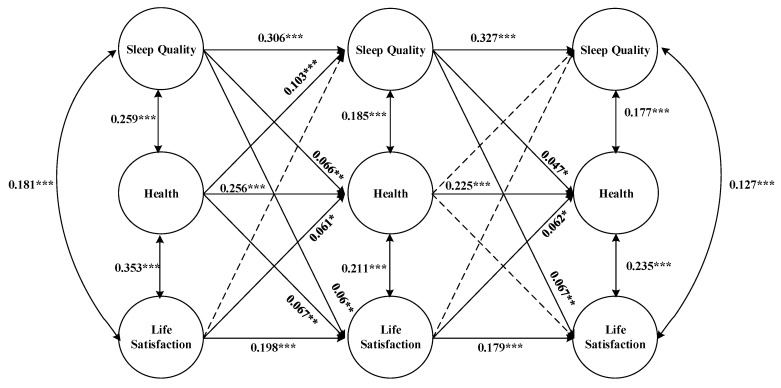
Mediating effect of health status on the reciprocal relationship between sleep quality and life satisfaction. Footnote: * denotes *p* < 0.05; ** denotes *p* < 0.01; *** denotes *p* < 0.001

As depicted in Figure 6, based on the introduction of control variables (age, gender, and marriage) and covariates (physical exercise, social security, and cognitive ability), the model fitting improved after incorporating health status as a mediator in the reciprocal association between sleep quality and life satisfaction, with fitting indicators of RMSEA = 0.045, CFI = 0.898, SRMR = 0.043, and *p* = 0.000.

In 2011, the sleep quality of older adults had a significant positive effect on their health status, life satisfaction, and sleep quality in 2014 (B = 0.306, *p* = 0.000; B = 0.06, *p* < 0.01; B = 0.066, *p* < 0.01, respectively). Similarly, the health status of the older population in 2011 had a significant positive effect on their health status, life satisfaction, and sleep quality in 2014 (B = 0.256, *p* = 0.000; B = 0.067, *p* < 0.01; B = 0.103, *p* = 0.000, respectively). However, the life satisfaction of older adults in 2011 did not significantly affect their sleep quality in 2014, but had a significant positive effect on their health status and life satisfaction in 2014 (B = 0.061, *p* < 0.05; B = 0.198, *p* = 0.000, respectively).

Similarly, in 2014, the sleep quality of older adults had a significant positive effect on their health status and life satisfaction in 2018 (B = 0.047, *p* < 0.05; B = 0.067, *p* < 0.01, respectively). However, the health status and life satisfaction of older people in 2014 did not significantly affect their sleep quality or life satisfaction in 2018, except for the significant positive effect of health status on health status (B = 0.225, *p* = 0.000). Moreover, the life satisfaction of the older population in 2014 did not significantly affect their sleep quality in 2018 but had a significant positive effect on their health status and life satisfaction in 2018 (B = 0.062, *p* < 0.05; B = 0.179, *p* = 0.000, respectively).

Furthermore, the sleep quality of older adults was significantly positively correlated with their health status and life satisfaction in all three time periods (2011, 2014, and 2018) (R ranging from 0.177 to 0.259, *p* < 0.01). Additionally, the health status of older adults was significantly positively correlated with their life satisfaction in all three time periods (R ranging from 0.211 to 0.353, *p* < 0.01).

## 4. Discussion

### 4.1. Analysis of the Trajectory of Sleep Quality, Life Satisfaction, and Health Status of Older Adults

There was clear evidence of a quadratic decline in sleep quality and health status but quadratic growth in life satisfaction across time, presented, respectively, in Figure 2, Figure 3 and Figure 4. The result of decreased sleep quality with age is in line with previous research [37,38]. The quality of sleep naturally changes with age, resulting in reduced duration and consolidation [39]. One plausible explanation is that, as people age, they are more prone to experiencing phenomena such as waking up early, waking up in the middle of the night, and having more nightmares [17,40], indicating sleep disturbance and thereby decreasing the sleep quality of older adults.

In this article, health status assessed by older adults themselves was reported to be associated with long-standing chronic illness [41,42], which is prone to occur in older adults due to exposure to chronic illness risk factors over their lifetime [43], thereby resulting in a decreased trend in health status, which is consistent with a previous study [27]. Also, from a biological standpoint, the health status of older individuals is influenced by age-related changes in muscles. As individuals age, muscles gradually lose their elasticity, leading to symptoms such as muscle weakness and muscle atrophy. These physiological changes significantly impact the overall health status of the older population [44].

In addition, for the change trajectory in life quality, extant studies demonstrate the U-shape relationship between age and life satisfaction from a life course perspective, indicating increased life satisfaction in later life [45,46,47], consistent with the research findings in this article. One plausible explanation is that individuals may adapt to their strengths and weaknesses as they age, leading to an increase in life satisfaction [48]. However, life satisfaction will gradually become stable and even decrease when reaching the oldest-old stage, due to the low level of perceived health [48].

### 4.2. Analysis of Reciprocal Relationship between Sleep Quality and Life Satisfaction

Figure 5 demonstrates a noteworthy reciprocal positive correlation between the sleep quality and the life satisfaction of older adults. A lagged effect exists for both sleep quality and life satisfaction. For instance, evidence suggests that the better the sleep quality, the better the life quality on the whole, which is consistent with extant studies [49,50]. In other words, poor sleep quality is linked with low life satisfaction [51]. As reported in a previous study, a single night of poor sleep quality has the potential to disrupt a person’s emotional well-being, leading to manifestations such as anger, depression, anxiety, and fatigue [52]. Also, poor sleep quality can have a detrimental impact on the enjoyment of daily life experiences [53]. Therefore, this study verified the positive cross-sectional association between sleep quality and life satisfaction. In addition, sleep quality during the previous period can predict life satisfaction during the subsequent period, indicating that sleep quality is not only associated with current life satisfaction but also with subsequent life satisfaction. Similar findings were found in a previous study [54]. One possible explanation is that poor sleep can have adverse effects on brain function, including impairments in cognitive processes, emotional regulation, and overall well-being. These neurophysiological disruptions can contribute to decreased subsequent life satisfaction over time [54].

In addition, the findings imply that higher life satisfaction is correlated with better sleep quality, which is in line with the previous study [16,49]. According to existing knowledge, individuals with low life satisfaction are more susceptible to experiencing anxiety and stress [52]. Moreover, anxiety and stress have been implicated as contributing factors to poor sleep quality [55]. However, there was no significant correlation found between life satisfaction in 2011 and sleep quality in 2014. However, it was found that life satisfaction in 2014 had a significant positive impact on sleep quality in 2018. This pattern suggests that life satisfaction in the middle-old period plays a vital role in sleep quality during the middle-old and oldest-old periods, indicating the lagged effect of life satisfaction on sleep quality existing during some periods, which is in line with a previous study [56].

### 4.3. Analysis of the Mediating Effect of Health Status in the Bidirectional Relationship between Sleep Quality and Life Satisfaction

According to Figure 6, the reciprocal relationship between sleep quality and life satisfaction remains significant even after introducing the mediator variable of health status. In addition, the mediating effect of health status between sleep quality and life satisfaction remains significant for every current period, 2011, 2014, and 2018.

Regardless of whether it is the variable of sleep quality, health status, or life satisfaction, the previous period has a significant impact on the subsequent period, indicating a discernible lagged effect on the variable itself. Furthermore, the time-lagged effect of sleep quality on life satisfaction remains significant, while the time-lagged effect of life satisfaction on sleep quality is no longer significant.

The lagged effect between sleep quality and life satisfaction remains valid after introducing the mediator of health status, indicating that health status plays a partial mediating effect in the relationship between sleep quality and life satisfaction. For instance, the direct lag effect of sleep quality in 2011 on life satisfaction in 2014 is 0.06 and the indirect lagged effect is 0.0139 (0.066×0.211). Similarly, the direct lag effect of sleep quality in 2014 on life satisfaction in 2018 is 0.067 and the indirect lag effect is 0.0110 (0.047×0.235).

However, the association between life satisfaction and sleep quality is completely mediated by the mediation of health status. For instance, the direct relationship between life satisfaction in 2011 and sleep quality in 2014 is not significant. The life satisfaction in 2011 impacted the health status in 2014, which affected sleep quality in the same year. This suggests that the lagged effect of life satisfaction on sleep quality is dependent on the mediating effect of health status, whereby the mediating effect is 0.0113 (0.061 × 0.185). Similarly, the direct lag effect is not significant between life satisfaction in 2014 and sleep quality in 2018, with the mediating effect being 0.0110 (0.062 × 0.177). In short, health status plays a crucial role as a partial or complete mediator in the reciprocal relationship between sleep quality and life satisfaction among older adults.

Research supports that sleep plays an important role in regulating internal metabolism and hormone levels, which are closely related to health status [57]. Also, sleep plays a crucial role in restoring the body, preserving energy levels, and maintaining overall health [16,58]. This implies that better sleep quality predicts better health status [57], while individuals with good health status are more likely to experience higher life satisfaction [7]. In addition, higher life satisfaction promotes the level of health status [27,28], thus increasing sleep quality [29]. Therefore, this can explain why health status plays a mediating role between sleep quality and life satisfaction.

## 5. Conclusions

The conclusions of this article are based on this research, which indicates that the sleep quality and health status of older adults experience a rapid decline over time, while their life satisfaction undergoes a gradual increase. Also, our study demonstrated the reciprocal positive relationship between sleep quality and life satisfaction and that the associations were either completely or partially mediated by the effects of the health status of older adults. To be specific, the higher the sleep quality, the better the health status, and the higher the life satisfaction. Also, the higher the life satisfaction, the better the health status, and the higher the sleep quality.

## 6. Contributions and limitations

This article utilizes longitudinal data for the first time to examine the trajectory of sleep quality, life satisfaction, and health status in older Chinese adults. By incorporating longitudinal data with the CLPA method, this study provides a deeper understanding of the temporal sequence and causal relationships between sleep quality and life satisfaction. Specifically, it allows for the examination of two competing hypotheses: whether one variable precedes the other or whether the association is bidirectional. In addition, it introduces a novel approach by utilizing three waves of data for conducting a mediation analysis. This advancement is significant as it goes beyond the common practice of examining statistical mediation solely through cross-sectional analyses. This methodological strength enhances the validity and robustness of the findings, making a valuable contribution to the existing research in this field. However, this study has some limitations as well. For instance, it exclusively employed health status as a mediating variable, but there could be additional mediating variables between sleep quality and life satisfaction, such as zero-sum beliefs [57]. Moreover, the evaluation of sleep quality in older adults is based on subjective reports provided by the elderly themselves, which may be deficient in terms of accuracy.

## 7. Implications

The bidirectional positive relationship between sleep quality and life satisfaction of older adults calls for the government and society to pay attention to the sleep quality and life satisfaction of the older population. Also, the partial or complete mediation effect between sleep quality and life satisfaction underscores the importance of the health status of older adults. Therefore, effective measures should be warranted to not only improve the sleep quality and life satisfaction but also the health status of older adults. Finally, the lagged effect of the three variables themselves further emphasizes the significance of sleep quality, health status, and life satisfaction at baseline in predicting future trends. To be specific, government departments have the potential to enhance public welfare by improving the quality of public facilities and services, thereby contributing to an overall increase in life satisfaction among older adults. In addition, it is imperative for physicians to offer sleep and health education to older adults and prioritize the diagnosis and treatment of sleep and health disorders, particularly among the high-aged older population. Moreover, as individuals age, there is a pronounced acceleration in a decline in sleep quality and health status. Thus, it becomes essential to strengthen sleep quality and health management and implement preventive measures during the initial stages of aging to mitigate the rapid deterioration of sleep quality and health status.

## Figures and Tables

**Table 1:** Definition of variables.

Variables	Definition	Mean (*n*%)
Age	Continuous variable (60~116)	80.52
Gender	Male = 1; female = 0	52%
Region	Urban = 1; rural = 0	8%
Marriage	Married = 1; others = 0	62%
Education	Years of education (0~20)	3.11
Smoking	Smoked = 1; never smoked = 0	35%
Alcohol drinking	Drunk = 1; never drunk = 0	30%
Physical exercise	Yes = 1; no = 0	35%
Social security	Number of social security (0~7)	1.37
Cognition ability	MMSE value (0~30)	26.38
Sleep quality	Continuous variable (1~5)	3.60
Health status	Continuous variable (1~5)	3.43
Life satisfaction	Continuous variable (1~5)	3.83

**Table 2:** Demographic analysis.

Variables	2011	2014	2018
Sleep quality	3.70	3.63	3.47
Life satisfaction	3.75	3.86	3.87
Health status	3.47	3.44	3.39
Age	77.22	80.11	84.23
Gender	0.52	0.52	0.52
Region	0.08	0.09	0.06
Years of education	3.13	3.11	3.08
Marriage	0.68	0.63	0.56
Social security	1.30	1.38	1.43
Physical exercise	0.41	0.35	0.30
Cognition ability	27.14	26.86	25.13

## Data Availability

Data derived from public domain resources.
